# 6,7-Dihydro-5*H*-1,4-diazepino[1,2,3,4-*lmn*][1,10]phenanthroline-4,8-diium tris­(thio­cyanato-κ*N*)cuprate(I)

**DOI:** 10.1107/S1600536810036779

**Published:** 2010-09-18

**Authors:** Na Xu, Yun-Yin Niu, Seik Weng Ng

**Affiliations:** a Department of Chemistry, Zhengzhou University, Zhengzhou 450052, People’s Republic of China; b Department of Chemistry, University of Malaya, 50603 Kuala Lumpur, Malaysia

## Abstract

The title copper(I) salt, (C_15_H_14_N_2_)[Cu(NCS)_3_], exists as non-inter­acting cations and trigonal–planar anions. The cation is buckled, the r.m.s. deviation of the atoms passing through the phenanthroline portion being 0.16 Å. The Cu^I^ atom is displaced by 0.019 (2) Å out of the N_3_ triangle. The crystal studied was a non-merohedral twin with twin domains in an approximate ratio of 55:45.

## Related literature

For a three-coordinate tris­(thio­cyanato)­cuprate(I) system, see: Song *et al.* (2008 ▶). For a study of the title cation, see: Liu *et al.* (2007 ▶).
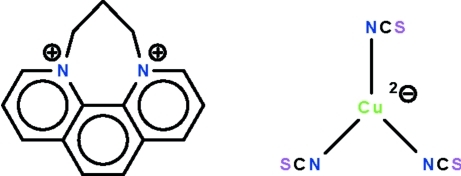


## Experimental

### 

#### Crystal data

(C_15_H_14_N_2_)[Cu(NCS)_3_]
*M* *_r_* = 460.06
Monoclinic, 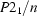

*a* = 17.2687 (4) Å
*b* = 6.5825 (2) Å
*c* = 17.2702 (4) Å
β = 107.803 (2)°
*V* = 1869.12 (8) Å^3^
*Z* = 4
Mo *K*α radiation
μ = 1.52 mm^−1^
*T* = 100 K
0.25 × 0.02 × 0.02 mm

#### Data collection

Bruker SMART APEX CCD-detector diffractometer
Absorption correction: multi-scan (*SADABS*; Sheldrick, 1996 ▶) *T*_min_ = 0.703, *T*_max_ = 0.970
15488 measured reflections
4303 independent reflections
3657 reflections with *I* > 2σ(*I*)
*R*_int_ = 0.048

#### Refinement

*R*[*F* ^2^ > 2σ(*F* ^2^)] = 0.034
*wR*(*F* ^2^) = 0.075
*S* = 0.99
4303 reflections
245 parameters
H-atom parameters constrained
Δρ_max_ = 0.38 e Å^−3^
Δρ_min_ = −0.37 e Å^−3^

### 

Data collection: *APEX2* (Bruker, 2009 ▶); cell refinement: *SAINT* (Bruker, 2009 ▶); data reduction: *SAINT*; program(s) used to solve structure: *SHELXS97* (Sheldrick, 2008 ▶); program(s) used to refine structure: *SHELXL97* (Sheldrick, 2008 ▶); molecular graphics: *X-SEED* (Barbour, 2001 ▶); software used to prepare material for publication: *publCIF* (Westrip, 2010 ▶).

## Supplementary Material

Crystal structure: contains datablocks global, I. DOI: 10.1107/S1600536810036779/zs2065sup1.cif

Structure factors: contains datablocks I. DOI: 10.1107/S1600536810036779/zs2065Isup2.hkl

Additional supplementary materials:  crystallographic information; 3D view; checkCIF report

## Figures and Tables

**Fig. 1.**
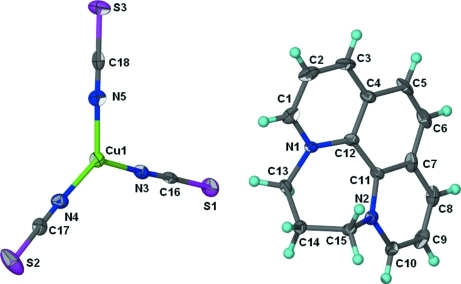
Displacement ellipsoid plot (Barbour, 2001) of [C15H14N2]2+ [Cu(NCS)3]2- at the 70% probability level.

## supplementary crystallographic information

### Comment

There is no report on a free, three-coordinate tris(thiocyanato)cuprate(I or II) ion in the structural literature. The potassium–benzene-18-crown-6 salt has the copper(I) atom in a three-coordinate environment but the sulfur ends of the thiocyanate ligands are also engaged in coordination (Song *et al.*, 2008). The title salt (Scheme I, Fig. 1) represents the first example of a three-coordinate trithiocyanatocuprate(I) system; the salt exists as discrete cations and anions. On the other hand, the cation has also been documented only once in the structural literature (Liu *et al.*, 2007). In the present salt, the phenanthroline portion is severely buckled (r.m.s. deviation of the plane passing through the atoms comprising the phenanthroline portion being 0.16 Å), with the nitrogen atoms deviating the largest distances (0.30, 0.30 Å).

### Experimental

6,7-Dihydro-5*H*-[1,4]diazepino[1,2,3,4-*lmn*][1,10]phenanthroline-4,8-diium] dibromide was synthesized by reacting 1,3-dibromopropane with 1,10-phenanthroline monohydrate. A methanol solution (10 ml) of the salt (0.40 g, 1 mmol) was mixed with a water/DMF (1:4) solution (10 ml) of colorless copper(I) thiocyanate (0.12 g, 1 mmol). An excess of potassium thiocyanate (0.50 g, 5 mmol) was added. The solution was filtered and the solvent allow to evaporate slowly to furnish dark brown crystals of the cuprate salt.

### Refinement

Hydrogen atoms were placed in calculated positions (C—H 0.95–0.99 Å) and were included in the refinement in the riding model approximation, with *U*_iso_(H) set to 1.2*U*_eq_(C).

### Crystal data

**Table:**

(C_15_H_14_N_2_)[Cu(NCS)_3_]	*F*(000) = 936
*M**_r_* = 460.06	*D*_x_ = 1.635 Mg m^−^^3^
Monoclinic, *P*2_1_/*n*	Mo *K*α radiation, λ = 0.71073 Å
Hall symbol: -P 2yn	Cell parameters from 2549 reflections
*a* = 17.2687 (4) Å	θ = 2.5–27.3°
*b* = 6.5825 (2) Å	µ = 1.52 mm^−^^1^
*c* = 17.2702 (4) Å	*T* = 100 K
β = 107.803 (2)°	Prism, brown
*V* = 1869.12 (8) Å^3^	0.25 × 0.02 × 0.02 mm
*Z* = 4	

### Data collection

**Table:**

Bruker SMART APEX CCD-detector diffractometer	4303 independent reflections
Radiation source: fine-focus sealed tube	3657 reflections with *I* > 2σ(*I*)
graphite	*R*_int_ = 0.048
ω scans	θ_max_ = 27.5°, θ_min_ = 1.2°
Absorption correction: multi-scan (*SADABS*; Sheldrick, 1996)	*h* = −22→21
*T*_min_ = 0.703, *T*_max_ = 0.970	*k* = −8→8
15488 measured reflections	*l* = −22→21

### Refinement

**Table:**

Refinement on *F*^2^	Primary atom site location: structure-invariant direct methods
Least-squares matrix: full	Secondary atom site location: difference Fourier map
*R*[*F*^2^ > 2σ(*F*^2^)] = 0.034	Hydrogen site location: inferred from neighbouring sites
*wR*(*F*^2^) = 0.075	H-atom parameters constrained
*S* = 0.99	*w* = 1/[σ^2^(*F*_o_^2^) + (0.0347*P*)^2^] where *P* = (*F*_o_^2^ + 2*F*_c_^2^)/3
4303 reflections	(Δ/σ)_max_ = 0.001
245 parameters	Δρ_max_ = 0.38 e Å^−^^3^
0 restraints	Δρ_min_ = −0.36 e Å^−^^3^

### Fractional atomic coordinates and isotropic or equivalent isotropic displacement parameters (Å^2^)

**Table:**

	*x*	*y*	*z*	*U*_iso_*/*U*_eq_	
Cu1	0.58165 (3)	0.28784 (6)	0.33388 (3)	0.01689 (10)	
S1	0.30831 (6)	0.32947 (13)	0.33202 (7)	0.0244 (2)	
S2	0.81220 (7)	0.20364 (14)	0.55830 (7)	0.0380 (2)	
S3	0.57925 (7)	0.23791 (13)	0.06016 (7)	0.0262 (2)	
N1	0.06515 (15)	0.3406 (4)	0.21244 (15)	0.0115 (5)	
N2	−0.03925 (18)	0.2372 (4)	0.31533 (17)	0.0138 (6)	
N3	0.47097 (19)	0.3174 (4)	0.33561 (18)	0.0180 (6)	
N4	0.67485 (17)	0.2767 (4)	0.42593 (16)	0.0194 (5)	
N5	0.58441 (19)	0.2614 (4)	0.2234 (2)	0.0184 (7)	
C1	0.1136 (2)	0.3304 (4)	0.1650 (2)	0.0181 (7)	
H1	0.1702	0.3554	0.1879	0.022*	
C2	0.0825 (3)	0.2840 (5)	0.0826 (2)	0.0215 (9)	
H2	0.1181	0.2658	0.0508	0.026*	
C3	0.0010 (3)	0.2649 (5)	0.0479 (2)	0.0222 (9)	
H3	−0.0208	0.2456	−0.0091	0.027*	
C4	−0.0516 (2)	0.2736 (4)	0.0965 (2)	0.0158 (8)	
C5	−0.1377 (3)	0.2680 (4)	0.0618 (3)	0.0219 (8)	
H5	−0.1611	0.2509	0.0048	0.026*	
C6	−0.1863 (3)	0.2870 (5)	0.1097 (3)	0.0224 (8)	
H6	−0.2434	0.2995	0.0856	0.027*	
C7	−0.1528 (2)	0.2885 (4)	0.1957 (2)	0.0181 (8)	
C8	−0.2032 (2)	0.2962 (5)	0.2473 (2)	0.0216 (9)	
H8	−0.2604	0.3089	0.2240	0.026*	
C9	−0.1704 (2)	0.2855 (5)	0.3296 (3)	0.0223 (9)	
H9	−0.2035	0.3044	0.3640	0.027*	
C10	−0.0880 (2)	0.2466 (4)	0.3622 (2)	0.0176 (8)	
H10	−0.0656	0.2261	0.4192	0.021*	
C11	−0.0685 (2)	0.2773 (4)	0.2317 (2)	0.0122 (7)	
C12	−0.0164 (2)	0.2952 (4)	0.1818 (2)	0.0136 (7)	
C13	0.09703 (17)	0.4419 (4)	0.29339 (17)	0.0145 (6)	
H13A	0.0576	0.5464	0.2984	0.017*	
H13B	0.1487	0.5116	0.2965	0.017*	
C14	0.11182 (19)	0.2948 (5)	0.3639 (2)	0.0163 (6)	
H14A	0.1165	0.3712	0.4145	0.020*	
H14B	0.1637	0.2221	0.3711	0.020*	
C15	0.04293 (18)	0.1418 (5)	0.34912 (18)	0.0143 (7)	
H15A	0.0499	0.0369	0.3107	0.017*	
H15B	0.0460	0.0735	0.4011	0.017*	
C16	0.4032 (2)	0.3236 (4)	0.3330 (2)	0.0137 (7)	
C17	0.7321 (2)	0.2479 (5)	0.4813 (2)	0.0168 (6)	
C18	0.5820 (2)	0.2508 (4)	0.1556 (2)	0.0164 (8)	

### Atomic displacement parameters (Å^2^)

**Table:**

	*U*^11^	*U*^22^	*U*^33^	*U*^12^	*U*^13^	*U*^23^
Cu1	0.0175 (3)	0.01500 (17)	0.0177 (3)	0.00063 (19)	0.00476 (13)	0.00035 (18)
S1	0.0145 (5)	0.0330 (5)	0.0246 (5)	0.0017 (4)	0.0041 (4)	−0.0004 (4)
S2	0.0355 (7)	0.0243 (5)	0.0366 (7)	0.0063 (4)	−0.0149 (4)	−0.0040 (4)
S3	0.0273 (6)	0.0356 (5)	0.0150 (5)	0.0062 (4)	0.0055 (4)	0.0010 (4)
N1	0.0122 (14)	0.0123 (12)	0.0096 (14)	0.0015 (10)	0.0027 (11)	0.0013 (10)
N2	0.0158 (15)	0.0101 (12)	0.0168 (15)	−0.0002 (10)	0.0068 (12)	−0.0003 (10)
N3	0.0205 (18)	0.0194 (15)	0.0140 (16)	−0.0013 (12)	0.0049 (13)	−0.0032 (11)
N4	0.024 (2)	0.0187 (14)	0.017 (2)	0.0009 (11)	0.0080 (9)	−0.0017 (11)
N5	0.0189 (17)	0.0179 (14)	0.0194 (18)	0.0047 (11)	0.0074 (14)	0.0004 (11)
C1	0.0185 (19)	0.0161 (17)	0.023 (2)	0.0050 (13)	0.0118 (15)	0.0040 (13)
C2	0.032 (2)	0.0166 (18)	0.021 (2)	0.0041 (15)	0.0149 (18)	0.0035 (13)
C3	0.040 (3)	0.0152 (15)	0.012 (2)	0.0002 (14)	0.0082 (18)	0.0010 (12)
C4	0.021 (2)	0.0113 (14)	0.0119 (19)	0.0009 (12)	0.0012 (15)	0.0008 (11)
C5	0.028 (3)	0.0134 (14)	0.014 (2)	−0.0009 (13)	−0.0087 (16)	0.0001 (13)
C6	0.018 (2)	0.0174 (14)	0.023 (3)	−0.0020 (14)	−0.0053 (16)	0.0002 (14)
C7	0.015 (2)	0.0088 (13)	0.029 (2)	−0.0001 (12)	0.0046 (16)	−0.0001 (13)
C8	0.013 (2)	0.0176 (15)	0.034 (2)	−0.0019 (13)	0.0062 (17)	−0.0001 (14)
C9	0.021 (2)	0.0186 (18)	0.034 (3)	−0.0001 (14)	0.0197 (19)	−0.0021 (15)
C10	0.023 (2)	0.0155 (16)	0.0165 (19)	−0.0054 (13)	0.0086 (16)	−0.0009 (12)
C11	0.0148 (19)	0.0092 (15)	0.0106 (18)	0.0025 (12)	0.0009 (14)	−0.0002 (12)
C12	0.0182 (19)	0.0094 (15)	0.0121 (18)	0.0014 (12)	0.0030 (14)	0.0031 (11)
C13	0.0133 (16)	0.0156 (15)	0.0133 (16)	−0.0012 (11)	0.0022 (12)	−0.0015 (11)
C14	0.0136 (19)	0.0207 (16)	0.0139 (19)	−0.0030 (12)	0.0032 (10)	0.0031 (12)
C15	0.0132 (15)	0.0165 (15)	0.0130 (16)	0.0020 (12)	0.0038 (13)	0.0043 (12)
C16	0.017 (2)	0.0129 (16)	0.0096 (17)	0.0021 (12)	0.0022 (13)	−0.0002 (11)
C17	0.019 (2)	0.0116 (17)	0.022 (2)	0.0000 (12)	0.0097 (11)	−0.0038 (13)
C18	0.0095 (18)	0.0114 (15)	0.027 (2)	0.0013 (11)	0.0027 (15)	0.0000 (12)

### Geometric parameters (Å, °)

**Table:**

Cu1—N4	1.886 (2)	C4—C5	1.423 (5)
Cu1—N3	1.930 (3)	C5—C6	1.353 (4)
Cu1—N5	1.930 (3)	C5—H5	0.9500
S1—C16	1.634 (4)	C6—C7	1.419 (6)
S2—C17	1.626 (3)	C6—H6	0.9500
S3—C18	1.637 (4)	C7—C11	1.400 (5)
N1—C1	1.340 (4)	C7—C8	1.424 (5)
N1—C12	1.378 (4)	C8—C9	1.361 (6)
N1—C13	1.494 (4)	C8—H8	0.9500
N2—C10	1.336 (4)	C9—C10	1.385 (6)
N2—C11	1.401 (4)	C9—H9	0.9500
N2—C15	1.497 (4)	C10—H10	0.9500
N3—C16	1.158 (4)	C11—C12	1.429 (4)
N4—C17	1.161 (3)	C13—C14	1.515 (4)
N5—C18	1.161 (5)	C13—H13A	0.9900
C1—C2	1.393 (5)	C13—H13B	0.9900
C1—H1	0.9500	C14—C15	1.519 (4)
C2—C3	1.356 (6)	C14—H14A	0.9900
C2—H2	0.9500	C14—H14B	0.9900
C3—C4	1.413 (5)	C15—H15A	0.9900
C3—H3	0.9500	C15—H15B	0.9900
C4—C12	1.418 (5)		
			
N4—Cu1—N3	125.77 (14)	C7—C8—H8	119.6
N4—Cu1—N5	123.83 (15)	C8—C9—C10	118.9 (3)
N3—Cu1—N5	110.37 (9)	C8—C9—H9	120.6
C1—N1—C12	120.7 (3)	C10—C9—H9	120.6
C1—N1—C13	118.3 (3)	N2—C10—C9	121.4 (4)
C12—N1—C13	119.9 (2)	N2—C10—H10	119.3
C10—N2—C11	121.4 (3)	C9—C10—H10	119.3
C10—N2—C15	118.7 (3)	C7—C11—N2	117.8 (3)
C11—N2—C15	118.7 (3)	C7—C11—C12	119.2 (4)
C16—N3—Cu1	175.2 (3)	N2—C11—C12	122.9 (4)
C17—N4—Cu1	172.8 (2)	N1—C12—C4	119.0 (3)
C18—N5—Cu1	176.2 (3)	N1—C12—C11	122.9 (3)
N1—C1—C2	121.2 (3)	C4—C12—C11	118.0 (4)
N1—C1—H1	119.4	N1—C13—C14	113.0 (2)
C2—C1—H1	119.4	N1—C13—H13A	109.0
C3—C2—C1	119.7 (3)	C14—C13—H13A	109.0
C3—C2—H2	120.1	N1—C13—H13B	109.0
C1—C2—H2	120.1	C14—C13—H13B	109.0
C2—C3—C4	120.2 (3)	H13A—C13—H13B	107.8
C2—C3—H3	119.9	C13—C14—C15	110.9 (2)
C4—C3—H3	119.9	C13—C14—H14A	109.4
C3—C4—C12	118.1 (3)	C15—C14—H14A	109.4
C3—C4—C5	121.8 (3)	C13—C14—H14B	109.4
C12—C4—C5	120.1 (3)	C15—C14—H14B	109.4
C6—C5—C4	120.2 (5)	H14A—C14—H14B	108.0
C6—C5—H5	119.9	N2—C15—C14	112.8 (2)
C4—C5—H5	119.9	N2—C15—H15A	109.0
C5—C6—C7	120.7 (5)	C14—C15—H15A	109.0
C5—C6—H6	119.7	N2—C15—H15B	109.0
C7—C6—H6	119.7	C14—C15—H15B	109.0
C11—C7—C6	120.0 (3)	H15A—C15—H15B	107.8
C11—C7—C8	118.4 (3)	N3—C16—S1	178.3 (3)
C6—C7—C8	121.6 (3)	N4—C17—S2	179.0 (3)
C9—C8—C7	120.8 (3)	N5—C18—S3	179.4 (3)
C9—C8—H8	119.6		
			
C12—N1—C1—C2	3.3 (5)	C15—N2—C11—C7	155.2 (3)
C13—N1—C1—C2	−164.4 (3)	C10—N2—C11—C12	171.5 (3)
N1—C1—C2—C3	5.5 (5)	C15—N2—C11—C12	−21.0 (4)
C1—C2—C3—C4	−5.8 (5)	C1—N1—C12—C4	−11.3 (4)
C2—C3—C4—C12	−2.2 (4)	C13—N1—C12—C4	156.1 (3)
C2—C3—C4—C5	175.7 (3)	C1—N1—C12—C11	171.5 (3)
C3—C4—C5—C6	−176.6 (3)	C13—N1—C12—C11	−21.1 (4)
C12—C4—C5—C6	1.2 (4)	C3—C4—C12—N1	10.7 (4)
C4—C5—C6—C7	−7.5 (4)	C5—C4—C12—N1	−167.2 (3)
C5—C6—C7—C11	2.2 (4)	C3—C4—C12—C11	−172.0 (3)
C5—C6—C7—C8	−176.2 (3)	C5—C4—C12—C11	10.1 (4)
C11—C7—C8—C9	−2.4 (5)	C7—C11—C12—N1	162.0 (3)
C6—C7—C8—C9	176.1 (3)	N2—C11—C12—N1	−21.8 (4)
C7—C8—C9—C10	−6.4 (5)	C7—C11—C12—C4	−15.2 (4)
C11—N2—C10—C9	3.6 (4)	N2—C11—C12—C4	161.0 (3)
C15—N2—C10—C9	−163.9 (3)	C1—N1—C13—C14	−111.1 (3)
C8—C9—C10—N2	5.9 (5)	C12—N1—C13—C14	81.2 (3)
C6—C7—C11—N2	−167.1 (3)	N1—C13—C14—C15	−42.1 (4)
C8—C7—C11—N2	11.4 (4)	C10—N2—C15—C14	−110.8 (3)
C6—C7—C11—C12	9.3 (4)	C11—N2—C15—C14	81.4 (3)
C8—C7—C11—C12	−172.2 (3)	C13—C14—C15—N2	−43.3 (4)
C10—N2—C11—C7	−12.3 (4)		
